# An Animated Functional Data Analysis Interface to Cluster Rapid Lung Function Decline and Enhance Center-Level Care in Cystic Fibrosis

**DOI:** 10.1155/2021/6671833

**Published:** 2021-05-10

**Authors:** Jesse Pratt, Weiji Su, Don Hayes, John P. Clancy, Rhonda D. Szczesniak

**Affiliations:** ^1^Division of Biostatistics & Epidemiology, Cincinnati Children's Hospital Medical Center, Cincinnati, OH, USA; ^2^Global Statistical Sciences Eli Lilly and Company, Indianapolis, IN 46285, USA; ^3^Division of Pulmonary Medicine, Cincinnati Children's Hospital Medical Center, Cincinnati, OH, USA; ^4^Department of Pediatrics, University of Cincinnati, Cincinnati, OH, USA; ^5^Cystic Fibrosis Foundation, Bethesda, MD, USA

## Abstract

Identifying disease progression through enhanced decision support tools is key to chronic management in cystic fibrosis at both the patient and care center level. Rapid decline in lung function relative to patient level and center norms is an important predictor of outcomes. Our objectives were to construct and utilize center-level classification of rapid decliners to develop an animated dashboard for comparisons within patients over time, multiple patients within centers, or between centers. A functional data analysis technique known as functional principal components analysis was applied to lung function trajectories from 18,387 patients across 247 accredited centers followed through the United States Cystic Fibrosis Foundation Patient Registry, in order to cluster patients into rapid decline phenotypes. Smaller centers (<30 patients) had older patients with lower baseline lung function and less severe rates of decline and had maximal decline later, compared to medium (30–150 patients) or large (>150 patients) centers. Small centers also had the lowest prevalence of early rapid decliners (17.7%, versus 24% and 25.7% for medium and large centers, resp.). The animated functional data analysis dashboard illustrated clustering and center-specific summaries of the rapid decline phenotypes. Clinical scenarios and utility of the center-level functional principal components analysis (FPCA) approach are considered and discussed.

## 1. Introduction

The analysis of patient registry data is an established cornerstone for epidemiologic surveillance of disease progression, assessment of treatment effectiveness, clinical trial design, and quality improvement initiatives [1]. The Cystic Fibrosis Foundation Patient Registry (CFFPR), which has been tracking outcomes on patients with cystic fibrosis (CF) for more than 40 years, successfully exemplified these processes [2]. As the most common lethal genetic disease in Whites, there are currently 30,000 individuals in the United States (US) and nearly 70,000 individuals worldwide living with CF [3, 4]. The disease typically manifests as progressive loss of lung function that results in respiratory failure; therefore, maintaining lung function is essential to quality of life and long-term survival. Modern survival statistics demonstrate that the advent of novel therapeutics and effective care management strategies have improved the longevity of individuals with CF; over half of babies born today with CF are projected to survive into their fifth decade of life [5]. Forced expiratory volume in 1 second of % predicted based on age, height, sex, and race (hereafter, FEV_1_) is the strongest predictor of survival [6] and the most widely used marker to monitor change in lung function over time for CF patients [7].

Despite gains in life expectancy, the clinical course of CF is still marked by periods of steep decline in FEV_1_ of variable patterns, primarily during adolescence and early adulthood [8]. This prolonged drop in lung function relative to patient or center-level norms, clinically termed “rapid decline,” has been heavily studied in recent years using various CF patient registries. Having real-time FEV_1_ trajectory data alongside information on risk factors for rapid decline significantly improved lung function in CF through empowerment of patients and families and standardizing care processes [9]. A host of risk factors for rapid decline in CF exist [10], with some being modifiable (e.g., number of clinic visits). To that end, quality improvement efforts by clinicians and researchers are focused on identifying the onset of rapid decline to intervene prior to onset of irreversible lung damage. Meanwhile, there are numerous epidemiologic studies using advanced biostatistical models demonstrating heterogeneity of lung function trajectories in the CF population. Timing and degree of rapid decline in lung function are highly variable between patients [11, 12]. Little research has characterized differences in rapid decline by CF center. Epidemiologic studies using CF registries in the United Kingdom (UK) and US found that centers with higher average lung function treated patients more frequently with intravenous antibiotics [13, 14].

In a single-center study, clinical algorithms successfully identified rapid declines as a means to trigger interventions and slow FEV_1_ loss by addressing modifiable risk factors, including untreated/newly identified infectious organisms, gaps in prescribed pulmonary therapies, and secondary diagnoses (e.g., gastroesophageal reflux disease) [15]. Follow-up studies of this algorithm showed that real-time risk assessment of rapid decline individualized at the patient level can be implemented in a graphical user interface prototype [16] and that this dynamic approach can detect this event an average of 8 months earlier [17]. Another single-center study developed a quality improvement algorithm to treat acute drops in FEV_1_, which resulted in an improvement in both absolute FEV_1_ (87% predicted to 110% predicted) and change in FEV_1_ (a drop of 34% predicted, compared to 14% predicted) over a 5-year period [18].

A national longitudinal cohort study using the CFFPR by our group established three phenotypes of rapid lung function decline in CF using longitudinal FEV_1_ trajectories in patients between 6 and 21 years of age [19]. By applying a functional data analysis technique known as functional principal components analysis (FPCA) and accounting for noisy, irregularly observed longitudinal FEV_1_ and serial correlation, three patient clusters were discovered corresponding to early, middle, and late rapid decline of lung function based on the first and third quartiles of the scores from the first functional principal component. Early decliners commonly experienced maximal loss around 12.9 years of age according to the average velocity of their FEV_1_ trajectories. Middle decliners, who comprised 50% of the cohort, had a peak decline at age 16.3 years. Late decliners had the mildest decline, which occurred at 18.5 years of age; however, this cohort experienced the most significant loss of lung function (roughly 6% predicted over the age range).

Despite the use of several algorithms for earlier detection and treatment to intervene to delay or prevent lung function decline, little has been done outside of descriptive reporting to leverage the CF data as indexed by individual care centers for improving care processes. The advent of such algorithms alongside a patient registry with electronic health records presents a unique opportunity, because both CF care and registry reporting are organized by center [3, 20]. This study aimed to (1) construct a center-level classification of rapid decliners in CF, expanding the prior approach which only classified decliner phenotypes at the national level; (2) utilize these novels, localized classifications of rapid decline to develop a graphical user interface for detection of decline, and comparison between centers, thereby enhancing point-of-care management.

## 2. Methods

### 2.1. Dashboard Design

**Figure 1:**
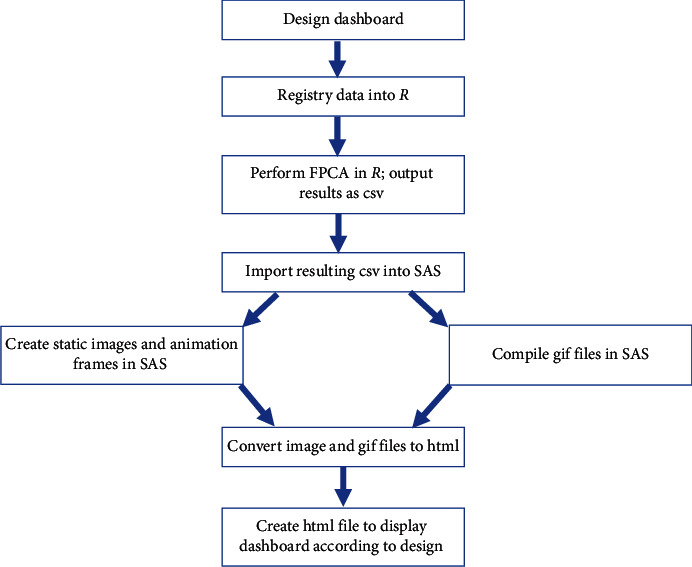
Dashboard design and implementation process.

Research methodology (Figure 1) proceeded as (1) designing the dashboard for center-specific benchmarking of rapid decline; (2) performing the classification approach in *R* software and importing results into SAS; (3) implementing the predictive data in SAS as a clinical dashboard. The dashboard was designed assuming downstream implementation on an html website platform. Preliminary layouts were developed by using images from the previously published study on phenotypes of rapid decline [19] and the format of the CFFPR Annual Report on center-specific outcomes [3].

### 2.2. Data Source

#### 2.2.1. Variables Used from the CFFPR

This study used the aforementioned CFFPR, which contains demographic and clinical variables from the majority of the CF population in the US who receive clinical care at accredited CF care centers. Data from clinical encounters and care episodes for each patient are included in the CFFPR. The timeframe for the current analysis was January 1, 1997, to December 1, 2013. Variables included age (in years) and lung function (FEV_1_, % predicted) at each clinical encounter. The % predicted values for FEV_1_ based on age, height, sex, and race were acquired using the Wang equations [21] for lung function on individuals aged 6 to 18 years and the Hankinson equations for those with encounter age older than 18 years [22]. Lung function data observed at encounter ages below 6 years were excluded because of potential unreliability [23]. Given the interest in rapid decline, which primarily occurs during childhood [8, 12, 24], the study included patients aged 6–21 years. Deidentified variables from the CFFPR were used to index both patients and centers. Center designation for each patient is recorded annually in the CFFPR. The center in which the patient spent the most years of follow-up over the analysis period was considered primary.

### 2.3. Missing Data

Patients with less than 7 observed FEV_1_ measurements were excluded, as having too few observations led to estimation difficulties. This study assumed that FEV_1_ data were “missing at random” [25], implying that one's ability to observe FEV_1_ on a given patient was related to their previously observed FEV_1_. Prior work found this to be a reasonable assumption for estimating CF lung function decline, especially in pediatric cohorts with low attrition due to death (4.1%) [26].

### 2.4. Clustering Approach

#### 2.4.1. Sparse FPCA

FEV_1_ measurements generated over time, expressed in this study as encounter age in years, may be considered longitudinal functional data. A branch of statistics known as functional data analysis offers a host of techniques to characterize curves resulting from these data [27]. FPCA is a particular functional data analysis technique used to cluster observed trajectories and acquire predicted values as smooth curves. Sparse FPCA works by segmenting variation in FEV_1_ trajectories in irregularly sampled longitudinal functional data according to eigenvectors, which correspond to functional principal components (FPCs) [27]. This approach utilizes a specialized estimation procedure known as restricted maximum likelihood [28]. Additional details on the approach are provided as supplemental material (Section S1).

### 2.5. Center-Level Aggregation

To conduct sparse FPCA, the “fpca” package in *R* was employed [29]. Implementation details are provided as supplemental material (Section S2). Each tracing was classified as corresponding to early, middle, or late decline based on the patient's score for the first functional principal component (denoted as FPC_1_). Higher scores coincided with more rapid decline. Patients with FPC_1_ scores less than the first quartile were considered late decliners; those with scores between the first and third quartiles were classified as middle decliners; finally, those whose scores exceeded the third quartile were considered rapid decliners. This quartile-based classification approach has been applied in prior biomedical studies [19, 30].

To estimate the center-level trajectory, the functional mean was taken across trajectories of patients who primarily received care at the given center according to their classified category of decline. Calculations are provided as supplemental material (Section S3). As a result of the computations, there were two data frames created in R. Both data frames were saved as csv files and imported into SAS v9.4 for html dashboard development.

### 2.6. Dashboard Implementation

#### 2.6.1. File Development

Once files from FPCA in *R* were imported into SAS, steps were taken to create html output for the clinical dashboard. Static images and animation frames were created using the SGPLOT procedure. Simultaneously, gif files were compiled using the SGPLOT procedure and ODS PRINTER destination. All image and gif files were converted to html format using the ODS HTML destination and DATA _NULL_ blocks containing PUT statements. To facilitate dashboard display, a total of nine centers were selected. Three centers per category were randomly chosen according to size (number of patients), defined as small (<30), medium (30 to 150), or large (>150).

### 2.7. Core Animations

A multistage sequence of transitions was programmed as animations for each center: (1) Given each patient's designation as an early, middle, or late decliner, their observed trajectories of FEV_1_ and rate of change over age were color-coded for the web tool as red, yellow, or green, respectively, for each type of image; (2) each observed FEV_1_ trajectory over age was converted to a smooth curve, ${\hat{f}}_{i}\left(t\right)$, meant to depict the true underlying lung function without measurement error; (3) within a given category of decline, the individual smooth curves were converted to the functional mean, ${\hat{f}}_{c\;d}\left(t\right)$. Notation for each function, *f*, aggregated is explained in the supplement (Section S3).

For the center-level rate-of-change animations, a similar sequence was programmed. The individual smooth curves representing lung function for patients receiving care at a given center, estimated as ${\hat{f\prime }}_{ic\;d}\left(t\right)$, were (1) presented on a graph; (2) shaded according to decline classification; (3) averaged within their given category of decline to yield three distinct estimated rate-of-decline curves.

### 2.8. Summary Curves

Thumbnail graphs showing center-specific functional means according to lung function trajectory were created. Similar graphs were generated for rate-of-change curves. Each set of graphics was generated to provide a snapshot of overall lung function and degree of rapid decline across centers. These results represented the end stage of the above core animations.

### 2.9. Web Page Structure

The html file was structured according to the preconceived dashboard design to include a landing page with distinct links for animated and summarized graphics of clinical interest. Links corresponded to center-level animations. A multimedia recording of the dashboard illustrating each of its segments is available upon request to the corresponding author.

### 2.10. Ethics

Cincinnati Children's Hospital Institutional Review Board approved the study [IRB: 2015–4518]. The requirement to obtain informed consent was waived given the study's retrospective nature. The CFFPR Committee provided data, which were stored on a password-protected, secure network. Patient and Center IDs from the CFFPR were deidentified and encrypted prior to data transfer. All CF Center IDs on the dashboard are deidentified, and no demographic or clinical data on patients are displayed, thereby minimizing reidentification risk. The dashboard has not been publicly launched online.

## 3. Results

### 3.1. Classifying Rapid Decline within Center

A total of 247 CF centers and 18,347 patients contributed data to our analysis cohort. Median center size was 52 patients, and the first and third quartiles of the size distribution were 21 and 100 patients, respectively. Patients receiving care at small centers tended to be older and experienced more rapid lung function decline (Table 1).

FEV_1_ = forced expiratory volume in 1 s of % predicted; FPC_1_ = first function principal component; FPCA = functional principal components analysis. ^*∗*^Results reported as *n* (%) or mean (SD) where the latter set of statistics is based on center-specific data obtained by averaging over patient-specific data. ^*∗∗*^Higher scores imply more rapid lung function decline. ^*∗*^Reported as mean (SD) of the percentages of each decline type across centers in a given category. Based on two-sample Welch *t*-test comparing center categories for each variable, *P* < 0.0001 for ^1^small versus medium; ^2^medium versus large; ^3^small versus large after Bonferroni adjustment; NS = not significant (statistically).

FPCA fit information is described in the supplement (Section S4). There were fewer early rapid decliners among the small centers, compared to medium and larger centers that had similar percentages of rapid decliners, on average. While mean prevalence of middle decliners per center was similar regardless of center size, there were proportionally fewer late rapid decliners at the medium and large centers, compared to small centers.

Peak decline analysis, performed post hoc using FPCA model fits, showed that patients typically experienced maximal loss at a slightly older age (about 6 months older, on average) than patients receiving care primarily at medium or large centers (Table 1). The extent of maximal decline, defined as the lowest point on the derivative of the fitted FEV_1_ trajectory, was also lower at the small centers, compared to the medium and large centers.

### 3.2. Clinical Dashboard Attributes

**Figure 2:**
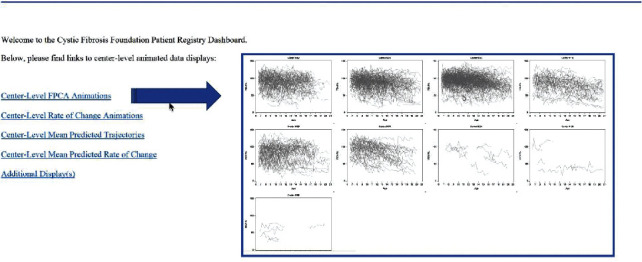
Landing page for dashboard with the blue arrow showing contents after clicking on the first link.

The landing page includes an overview of links with the first four links showing separate graphs by center (Figure 2). A 5^th^ link was created for additional displays, in the event that extra animations or other displays are developed. A point-and-click video of the dashboard display is available as previously mentioned by request to the corresponding author. A link to view a video file of the animation is provided in the supplement (Section S5).

### 3.3. Animated FPCA of Lung Function Trajectories

**Figure 3:**
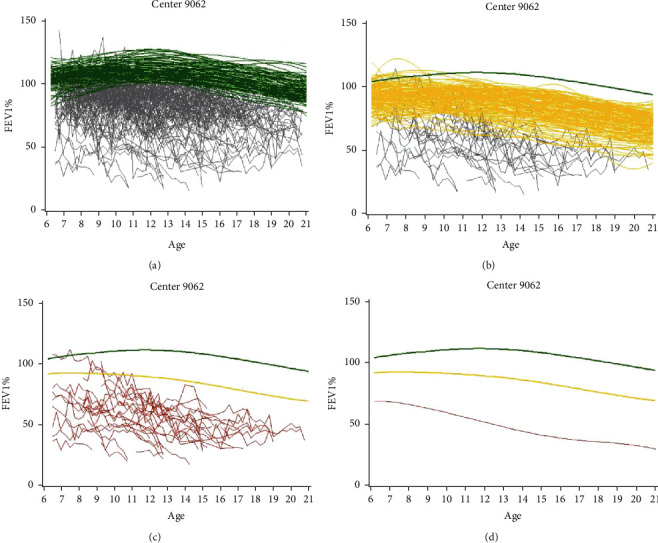
Predicted lung function trajectories by center clustered using FPCA phenotypes and animated on the dashboard (screenshots). Animation sequence shown for an example center on the interface beginning clockwise: (a) the clustering and smoothing of late decliners (green); (b) middle decliners (yellow); (c) early decliners (red); (d) finalized and smoothed phenotypes. Full animation available from link “Center-Level FPCA Animations” on interface.

The link “Center-Level FPCA Animations” contains scatterplots from the nine selected centers (Figure 2, center scatterplots). The opening segment of the link demonstrates the variability at multiple levels. There is substantial variability between patients and within an individual patient over time. Furthermore, there is heterogeneity between centers as depicted by the multiple graphic tiles. Clicking on any single tile containing a center's graph isolates the dashboard to viewing only the observed FEV1 trajectories from that center. At that point, the animation begins with highlighting individual observed trajectories from late decliners, smoothing those trajectories into fitted curves (green lines, Figure 3(a)), and collapsing them into a single mean curve. Once trajectories are highlighted, the variability between patients who are within the same type of decline is still noticeable. Next, the middle decliners' trajectories are classified using the same process (yellow lines, Figure 3(b)), followed by the trajectories of early decliners (Figure 3(c), observed trajectories shown in red prior to smoothing). The resulting three mean profiles of rapid decline for early, middle, and late groups are shown as the final result of the animation (Figure 3(d)). Each animated graph shows the observed and predicted FEV_1_ over age (in years).

### 3.4. Rate-of-Change Animations

**Figure 4:**
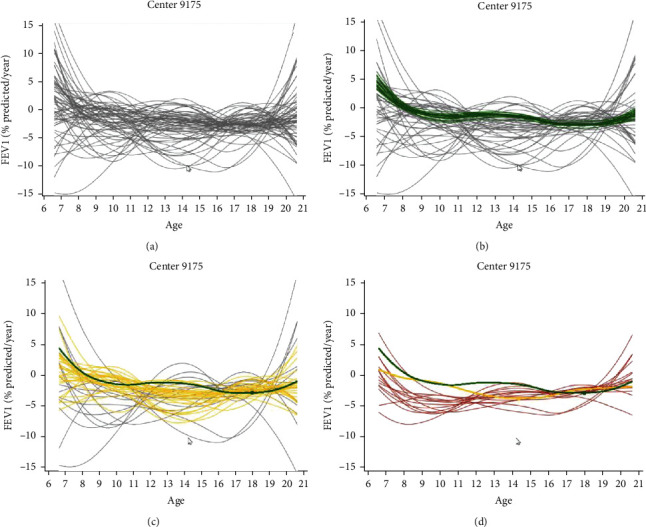
Rate-of-change curves projected and clustered by FPCA phenotypes, animated on dashboard (screenshots). Animation sequence shown for an example center on the interface beginning clockwise with (a) showing scatterplot of smoothed curves prior to clustering in gray; (b) late decliners already clustered (green curve) and initial clustering of middle decliners (yellow curves); (c) early decliners (red); (d) finalized and smoothed phenotypes. Full animation available from link “Center-Level Rate-of-Change Animations” on interface.

This set of animations, shown under the link “Center-Level Rate-of-Change Animations,” demonstrates how clustering of rapid decline is achieved and how this attribute is assessed with a rate of change of the smoothed FEV_1_ trajectories. Consistent with the link above, clicking this link leads to an interface displaying the smoothed rate-of-decline curves observed across centers, where each center is represented with a distinct graph of rate of change in FEV_1_ (% predicted per year) over age (in years). There is also variability between patients with regard to the smoothed curves, as well as between centers. The clustering process for a selected center graph begins with individual smoothed curves of the rate of change in FEV_1_ trajectories (Figure 4(a), gray curves). Animation proceeds first by collapsing the curves of late decliners into a single mean curve; hence, gray curves are being collapsed (Figure 4(b), also showing collapsing green curves). Then, yellow curves are highlighted and clustered into a single mean curve (Figure 4(c)). Finally, red curves of early decliners are highlighted and clustered (Figure 4(d)), eventually resulting in three separate curves corresponding to early, middle, and late.

### 3.5. Static Graphical Summaries

**Figure 5:**
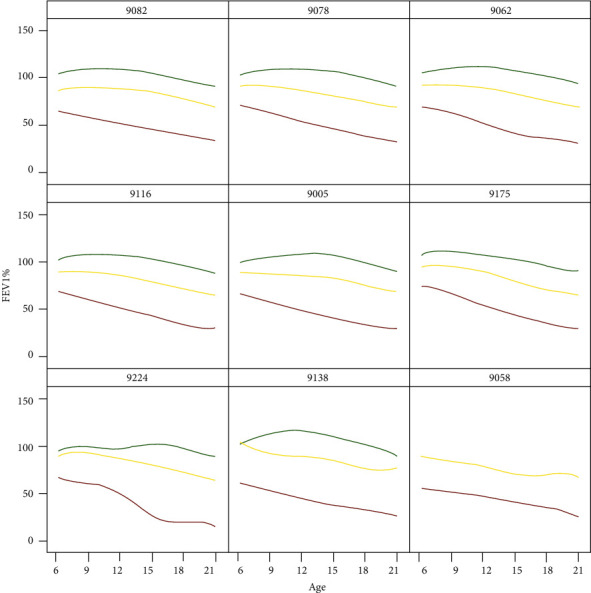
Predicted lung function trajectory classified by FPCA phenotype shown according to select centers. The smoothed mean curves represent phenotypes of rapid decline according to early (red), middle (yellow), and late (green). Static image taken from link “Center-Level Mean Predicted Trajectories” on the interface.

**Figure 6:**
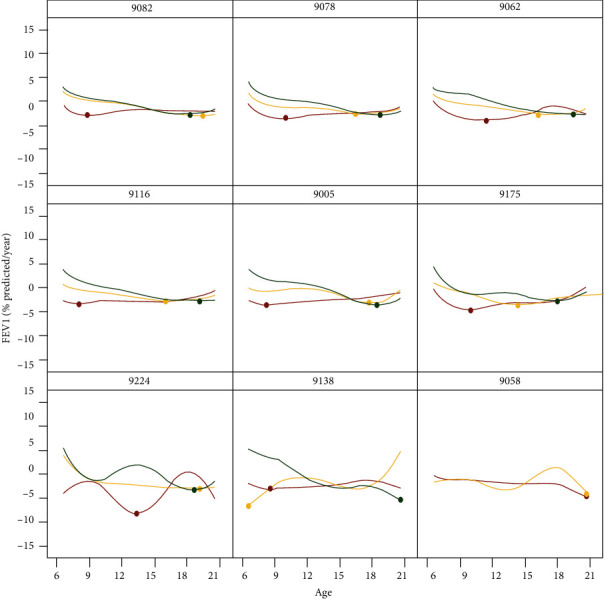
Predicted rate of decline in lung function trajectory classified by FPCA phenotype shown according to select centers. The smoothed mean curves represent phenotypes of rapid decline according to early (red), middle (yellow), and late (green). Static image taken from link “Center-Level Mean Predicted Rate of Change” on the interface.

Aligned with previously described links for the observed FEV_1_ trajectory and rates of change, summary links are available through the dashboard. These links circumvent the animation processes, in the event that a clinician prefers an immediate overview of the average trajectories across centers. The link “Center-Level Mean Predicted Trajectories” includes the smoothed average trajectories of lung function corresponding to early, middle, and late rapid decliners (Figure 5). The rows are organized by center size, so that the top, middle, and bottom rows coincide with small, medium, and large centers, respectively. Similarly, the link “Center-Level Mean Predicted Rate of Change” is set up to show a snapshot of the severity of decline based on the rate of change in lung function (Figure 6). The curves indicate increased fluctuation in decline for the smaller centers, which have fewer patients. For example, one of the small centers has no late decliners, as depicted by the absence of a green curve.

### 3.6. Benchmarking Centers to Assess Rapid Decline

For purposes of quality improvement or patient-centered care, the dashboard facilitates clinically relevant benchmarking of rapid decline through assessments of individual patient progression, patient trajectories within a single center, and summarized trajectories between centers. Clinically relevant scenarios are provided (Table 2).

## 4. Discussion

This is the first study to translate a statistical model for center-level assessment of rapid disease progression in individuals with CF. Prior CF studies enabled strides in clinical research and care by demonstrating that lung function could be improved through implementation of FEV_1_-guided decision rules. For example, the rule instituted by McPhail and colleagues required that the patient experience at least a 10-point drop from the maximum FEV_1_ observed in the past year before being classified as a rapid decliner [9, 15, 31]. Schechter's study instituted a stratified quality improvement algorithm, wherein slight (<5 point) decreases in FEV_1_ from baseline warranted routine therapy and follow-up; decreases from 5 to 10 points with no change in symptoms would lead to adherence assessments and more stringent follow-up; new signs and symptoms or FEV_1_ drop exceeding 10 points signaled adherence assessment, antibiotics regimens, and more aggressive follow-up [18].

Decision rules from these past studies have been shown to be effective but are aimed at intervening on lung function loss once it has already occurred. The proposed approach with FPCA provides predicted FEV_1_ trajectories, which could enable risk assessment of future lung function decline, thereby allowing proactive patient-focused management. Of statistical and ultimately clinical relevance is the reduction of measurement error allowed by mixed-modeling approaches [32], for which FPCA is an example. Measurement error is a well-studied issue with pulmonary function testing when collecting FEV_1_, showing that this measure of lung function varies considerably within a single testing session [33]. Indeed, FEV_1_ variability is an emerging marker of CF lung disease severity [34].

Another important distinction of our work is that previously published decision rules and accompanying algorithms have been limited to their centers of origin, which can hinder generalizability and add center bias. Our comparative analysis by center size indicates that small centers may have more frequent rapid decline, yet the average peak decline is lower given the older ages at these centers, compared to larger-size centers (Table 1). While our study focused on a pediatric cohort, the approach could be extended to monitor adult CF lung function trajectories. Although the most rapid decline tends to occur in adolescence and early adulthood [8], bouts can be experienced throughout the lifespan [17]. Our clinical dashboard enables comparison between centers, offering the opportunity for accelerated, multicenter quality improvement research while reducing center-specific bias that may exist. Real-time assessment of lung function trajectories with this dashboard could be integrated into electronic medical record systems as healthcare centers. These implementations may be useful for previsit planning processes and monitoring of home spirometry data that are becoming more commonplace in CF care as a result of COVID-19 impacts to outpatient visits [35]. The dashboard could be updated at a given center with electronic medical record systems through weekly updating, for example, every Sunday evening, which would enable up-to-date projection for outpatient visits scheduled for the coming week.

Findings from this study on the feasibility of a multicenter dashboard complement ongoing efforts of the Cystic Fibrosis Foundation to establish quality improvement initiatives and a learning network across a consortium of CF Care Centers in the US, motivating European efforts [36]. Using a novel approach characterizing the timing and extent of rapid lung function decline across CF centers, our dashboard is innovative in promoting the portability of interventions across the CF center network. While a recently published study provides a dashboard and retrospective validation for forecasting rapid lung function decline using the CFFPR, the modeling approach and resulting dashboard do not account for center variation or cluster rapid decline [17]. While the FPCA approach has established rigor, the animated dashboard developments are preliminary and require additional input from clinicians and patients prior to implementation and future research to further validate our current findings. These inputs will be vital to optimizing provider utility and share decision-making between the clinician and patient.

An important factor in rapid lung function decline in the CF population is the contribution of the pulmonary exacerbation. Without a consensus on the definition, the contribution of the CF pulmonary exacerbation is poorly understood. This acute respiratory event can permanently lower an individual's lung function trajectory [37]. Clinical diagnosis criteria for a pulmonary exacerbation widely vary, but an acute reduction in FEV_1_ is universally present [38]. Our approach allows assessment of the rate of change in FEV_1_ over the continuum of age, enabling clinicians to select the extent and timing of rapid decline for intervention (Figure 4). Furthermore, the missing-at-random assumption within the FPCA applied here using the mixed model framework accounts for irregular sampling bias [39]. In this context, the bias refers to patients who are “sicker” as defined by clinical characteristics being more likely to have frequent encounters, compared to other patients. In turn, these patients contribute to more FEV_1_ and other data, which can yield misleading study results in the form of misclassifying rapid decliners. The use of our dashboard may further categorize rapid decliners according to the frequency and severity of pulmonary exacerbations, but that requires further study. As previously discussed with dynamic updating, accruing new data on a given patient would enable continued classification of their rapid decline status.

Findings from this study have important implications for CF clinical research and care and other chronic diseases. Perhaps the most prominent is the ability to further mitigate Type I and Type II errors of rapid decline interventions (Table 3). The dashboard could aid in avoiding unnecessary exposure to antibiotics, for example, as decisions could be made according to the rate of change in the patient's trajectory, thereby minimizing Type I errors (Table 3, upper right quadrant with false positives). Incorrectly deciding that a patient is not experiencing rapid decline and foregoing treatments could lead to irreversible lung damage; a Type II error that could be decreased (Table 3, lower left quadrant with false negatives). As a corollary to this result, examining a single patient's lung function trajectory alongside overall severity profiles at the center level could improve personalized intervention while maintaining quality improvement goals. For example, a center's care episode capacity could be examined against the total number of patients allocated for intravenous antibiotics hospitalization according to the algorithm. The dashboard presented here could be used to compute projected totals, allowing the center care team to balance sensitivity and specificity as part of determining a cut point or, more specifically, their patients' FPC_1_ scores (Table 1) or degree of rapid decline projected at a given time (Figure 4). The process outlined here could be adapted for other chronic diseases and disorders relying on observing longitudinal outcomes, such as glycemic control and management for patients with type 1 diabetes, an area in which FPCA has been applied but not translated for dashboard development or implementation [30, 40]. Another implication is the potential utility of this approach for selecting subpopulations within centers who may benefit from new treatments. Ninety-one CF care centers recorded through the CFFPR are clinical research centers belonging to the Therapeutics Development Network; thus, the dashboard could optimize clinical trial planning and recruitment according to rapid decline severity.

This work has several limitations. Although FEV_1_ is a prominent surrogate endpoint [6] and past values alone accurately predict CF disease progression [17], there are established risk factors for rapid decline, such as biological sex and respiratory infections [10], as well as secular changes that are typically accounted for by including a covariate for birth cohort [12], not studied that could be incorporated into FPCA to perform supervised clustering of FEV_1_ trajectories. This FPCA implementation does not include measures of variability around the estimated curves, but this could be added in the future as confidence intervals by conducting bootstrapping as in prior work [19]; these could be constructed for both the absolute and rate-of-change trajectories. Current methods, however, are still in experimental stages, for example, allowing only one covariate/factor at a time [41], which would be critical so that the impact of highly effective modulator therapies could be considered a covariate effect [42, 43]. A recent update aimed at covariate adjustment on the marginal covariance in FPCA, enabling more efficient estimation in instances with several covariates [44], but the extension in the context of longitudinal data was not addressed. A notably difficult problem with rapid decline classification is the lack of a “gold standard” when using rate-of-change estimates as in the current study. Many past studies have relied on the occurrence of an intravenous hospitalization for pulmonary exacerbation diagnosis in retrospective modeling, such as a recent joint longitudinal FEV_1_ and exacerbation model [45], but this approach does not consider the variability involved in making such a diagnosis. An alternative is to utilize short-term drops in FEV_1_ on the original scale of % predicted, but it does not account for measurement error and lacks interpretation as rapid decline since it is not expressed as % predicted/year. The data presented with this dashboard were sampled from 1997 to 2013 prior to the broad initiation of modulator therapies that treat the underlying defect of CF. However, the dashboard generated from our study could be updated with new data as it is implemented for real-time monitoring. Trajectory levels may also change if using Global Lungs Initiative (GLI) equations to calculate ppFEV1 but the impact on rate-of-decline estimates appears to be minimal [46]. Finally, the current dashboard utilized deidentified and encrypted IDs. Some degree of unblinding would be necessary for real-world implementation and between-center testing.

## 5. Conclusion

We clustered and predicted rapid lung function decline at the center level by adapting a novel statistical approach. Moreover, we implemented our approach as a dashboard that can enhance quality improvement efforts through center benchmarking and research by identifying at-risk subpopulations for new treatments aimed at improving rapid decline. Our study findings and clinical dashboard with animated FPCA clustering provide a basis for chronic disease management in CF with the potential translation of this technology to other patient populations who may benefit from personalized management of rapid disease progression.

## Figures and Tables

**Table 1:** Features of rapid lung function decline summarized by primary care center^*∗*^.

Features	Center type		
	Small (<30)	Medium (30–150)	Large (>150)
Center characteristics			
No. of centers, *n* (%)	78 (31.6%)	133 (53.8%)	36 (14.6%)
No. of patients per center	13.3 (8.1)	72.7 (31.0)	213.3 (60.5)
No. of patients across centers	1036 (5.6%)	9674 (52.6%)	7677 (41.8%)
Patients within centers			
Age at baseline, years	13.5 (2.9)^1^	10.0 (1.8)^2^	8.6 (0.5)^3^
FEV1 at baseline, % predicted	75.7 (13.9)^1^	82.9 (6.3)^2^	85.6 (5.2)^3^
Trajectories			
FPCA			
FPC_1_ score^*∗∗*^	5.3 (10.9)^1^	2.3 (5.7)^2^	1.1 (5.3)^3^
Decline types by center, %^*∗∗∗*^			
Early	17.7 (17.1)^1^	24.0 (20.7)^2^	25.7 (22.7)^3^
Middle	49.9 (9.7)^NS^	49.8 (7.6)^NS^	49.7 (9.9)^NS^
Late	32.4 (9.1)^1^	26.2 (6.6)^2^	24.6 (8.2)^3^
Peak decline			
Extent, % predicted/year	−5.8 (1.3)^1^	−6.5 (0.7)^2^	−6.4 (0.6)^3^
Age of occurrence, years	15.6 (2.2)^1^	15.1 (0.8)^2^	15.0 (0.5)^3^

**Table 2:** Point-of-care scenarios and animated FPCA dashboard utility.

Scenario	Decision support offered by dashboard
Small center clinician benchmarking	Clinicians at Center 9138, one of the smaller centers, would like to examine the extent of rapid decline at their center and compare it to a larger center, for example, Center 9078. The clinicians can acquire an overview of these two centers on the static images' link of the dashboard (Figure 6). While the late decline phenotype appears similar at the centers (green curves), there are differences between centers with respect to both early and middle decliners (red and yellow curves, respectively). The clinicians can also compare their center to other centers. Variability at each center may be examined by selecting the first link on the landing page and selecting animation for the smaller center and any comparative centers.
Patients who receive care within a single large center	For clinicians and care teams at a larger center, such as Center 9062, it may be of interest to examine trajectories of lung function for patients within their center and how they cluster according to rapid decline. Clinicians can select the graphics tile corresponding to their center using the first link on the landing page.
Identifying at-risk patients for implementation research	For researchers preparing to initiate a new algorithm to improve lung function at their center, the dashboard could be used to select patients at the highest risk of rapid decline by examining the profiles and prevalence of early decliners within a given center. Furthermore, the dashboard could be used to examine the prevalence of early decliners across centers, if the researchers are planning to implement the algorithm at multiple sites. For this case, the dashboard enhances comparisons between centers.
Individual patient monitoring	At a single center, a clinician is preparing for outpatient visits in the coming week. The dashboard could be used to track his specific outpatients and their status as early, middle, or late decliners. Those classified by the algorithm as early decliners could be tagged and additional care regimens could be implemented, for example, psychosocial assessments and checking adherence [15]. It is also possible that these patients could be offered clinical trial participation based on their trajectories.

FPCA = functional principal components analysis.

**Table 3:** Clinician decision-making and consequences regarding rapid decline at the center level.

Condition of rapid decline (true underlying state)				
		Positive	Negative	Total
Clinician decision (diagnosis)	Positive	True positive (TP): correctly classified as rapid decliner	False positive (FP): incorrectly classified as rapid decliner	TP + FP = total number of patients allocated for rapid decline intervention
	Negative	False negative (FN): incorrectly classified as not experiencing rapid decline	True negative (TN): correctly classified as not experiencing rapid decline	FN + TN = total number of patients who will not receive rapid decline intervention
	Total	TP + FN = true number of rapid decliners	FP + FN = true number of patients not experiencing rapid decline	N = total number of patients at the care center

## Data Availability

Under an information use agreement with the Cystic Fibrosis Foundation, the authors are not permitted to share the data used in this study. Requests regarding these data may be emailed to the Cystic Fibrosis Foundation: datarequests@cff.org.

## Supplementary Materials

Supplementary Materials Supplementary material is available at the *Journal of Healthcare Engineering* online. We include an overview of sparse functional principal components analysis, details of the implementation of the analytic approach, how we calculated center-level aggregated values of rapid decline parameters, and how we performed model selection and there is a description of the link to our dashboard demo: https://confluence.research.cchmc.org/display/RESPUB/Animated+CF+Center-Level+FPCA.
