# Supplementary material for: An Animated Functional Data Analysis Interface to Cluster Rapid Lung Function Decline and Enhance Center-Level Care in Cystic Fibrosis
Source: J Healthc Eng. 2021 May 10;2021:6671833. doi: 10.1155/2021/6671833 (PMC8140832; doi:10.1155/2021/6671833)
Supplement: Supplementary Materials — Supplementary material is available at the Journal of Healthcare Engineering online. We include an overview of sparse functional principal components analysis, details of the implementation of the analytic approach, how we calculated center-level aggregated values of rapid decline parameters, and how we performed model selection and there is a description of the link to our dashboard demo: https://confluence.research.cchmc.org/display/RESPUB/Animated+CF+Center-Level+FPCA. [file 6671833.f1.docx]

**Supplement to “An Animated Functional Data Analysis Interface to Cluster Rapid Lung-Function Decline and Enhance Center-Level Care in Cystic Fibrosis”**

Jesse Pratt, Weiji Su, Don Hayes Jr, John P. Clancy, Rhonda D. Szczesniak

**S1. Overview of** **sparse functional principal components analysis (FPCA)**

The general concept of FPCA similar to that of principal components analysis (PCA), which maximizes total variation among a set of multivariate data according to eigen values corresponding to principal components. Practical explanations of FPCA as a functional extension of PCA have been provided([28](#_ENREF_28)). In addition to predicted values along smoothed individual trajectories, FPCA also yields scores for each functional principal component.

Sparse FPCA performs well for mistimed repeated measurements([29](#_ENREF_29)), which is the nature of how FEV_1_ is observed in people with CF. Patients attend clinical encounters either for well visits or when experiencing mild clinical manifestations of the disease. For severe symptoms, patients are hospitalized, and lung function may be collected during the care episode. Attrition occurs through the lifespan in these trajectories due to death.

**S2. Implementation of sparse FPCA**

To improve the convergence and efficiency of the algorithm, the variable age was scaled between 0 and 1. Once FPCA was completed, age was back transformed to the original scale for generating predicted values. A suitable basis function for representing the eigenfunctions was chosen using cubic B-splines. The “fpca” package implements restricted maximum likelihood estimation through a Newton-Raphson procedure, in order to estimate the functional principal components (FPCs) from the FEV_1_ data. This approach addressed the selection of the number of basis functions, as well as the dimension of the process (i.e., number of nonzero eigenvalues) used in the model by minimizing an approximation of the leave-one-curve-out cross-validation score. The algorithm was implemented using patient-specific quarterly mean FEV_1_ (expressed as % predicted) as the response variable. Cubic B-splines were specified with equally spaced knots. The candidate models had different settings of $M$ and $r$, which represented the number of basis functions for the eigenfunctions and the dimension of the process (number of nonzero eigenvalues) used in the model. Combinations of 𝑀 = (4,5,6,8) and 𝑟 = (2,3,4,5) were examined, and results were reported from the model with the best (i.e., the smallest) cross-validation score.

**S3. Calculations for center-level aggregation of FPCA**

Using the basis functions and coefficients from the best fitting FPCA model, individualized tracings were obtained for all patients used in the analysis. An equally spaced grid of time was formed to represent encounter age (in years), and time points from this grid were denoted as $t$, where $t\in(6, 21)$. The functional mean, defined from functional data analysis methodology(1), for each center was computed as follows.

$$\hat{f}_{cd}\left( t \right)=\frac{1}{N_{cd}}\sum_{i=1}^{N_{cd}} \hat{f}_{icd}\left( t \right),$$

where $\hat{f}_{icd}\left( t \right)$ is the function of principal component scores estimating the FEV1 trajectory of the $i^{th}$ patient within the $c^{th}$ center $(c=1,\ldots,C)$ according to their decline classification of early, middle or late $(d=1,2,3)$; $N_{cd}$ represents the total number of patients at the $c^{th}$ center with decline classification $d$. The center-specific functional mean, estimated as $\hat{f}_{cd}$, is evaluated over time $t$ expressed as encounter age (in years). The estimated rate of lung function progression for each center, $\hat{f'}_{cd}\left( t \right)$, was obtained by numerically approximating the derivative over the aforementioned grid. The “diff” command in R was used to compute the derivatives corresponding to rate of progression.

The calculations are part of the two data frames created in R. Included are the variables used as inputs for FPCA and the resulting smoothed function and derivative estimates for each patient ($\hat{f}_{icd}\left( t \right)$ and $\hat{f'}_{icd}\left( t \right)$), and the second data frame has the computed functional means and derivatives specific to center ($\hat{f}_{cd}\left( t \right)$ and $\hat{f'}_{cd}\left( t \right)$). The categorical variable $d$ classifying each patient as an early, middle or later decliner was added to each data frame.

**S4. Model selection results from FPCA**

The optimal FPCA model had $M=6$ and $r=5$ for the number of basis functions for the eigenfunctions and the dimension of the process, respectively. The FPCA variance proportions for the five functional principal components retained were 0.9397, 0.0347, 0.0155, 0.0055 and 0.0047, respectively. Thus, the first functional principal component (FPC_1_) explained roughly 94% of the total variation among the FEV_1_ trajectories.

**S5. Accessing demo of dashboard for center-level FPCA**

The link below provides access to a brief .mov file reviewing functionality of the animated dashboard:

<https://confluence.research.cchmc.org/display/RESPUB/Animated+CF+Center-Level+FPCA>

Inquiries regarding the link or app may be sent to the corresponding author, Rhonda Szczesniak. Email: [rhonda.szczesniak@cchmc.org](mailto:rhonda.szczesniak@cchmc.org)

**References**

1. Ramsay JO, Silverman BW. Functional data analysis. 2nd ed. New York: Springer; 2005. xix, 426 p. p.
